# Long-Term Survival After Repeated Salvage Chemoradiation Therapy for Metastatic Lymph Node Recurrence in Advanced Gastric Cancer: A Case Report

**DOI:** 10.7759/cureus.36649

**Published:** 2023-03-24

**Authors:** Atsuto Katano, Hideomi Yamashita

**Affiliations:** 1 Radiology, The University of Tokyo Hospital, Tokyo, JPN

**Keywords:** multimodal treatment, recurrence, long-term survival, salvage chemoradiation therapy, chemotherapy, gastric cancer

## Abstract

Metastatic recurrence in advanced gastric cancer is a poor prognosis, and where recently novel systemic therapies have been investigated. This case report describes the successful use of repeated salvage chemoradiation therapy in a patient with advanced gastric cancer who had failed initial treatments. The patient achieved long-term survival and remained disease-free for several years after treatment. The report highlights the potential benefits of salvage chemoradiation therapy in selected patients with advanced gastric cancer and the need for further studies to determine the optimal treatment approach for these patients. The report also discusses recent promising results from clinical trials of combination regimens with immune checkpoint inhibitors and targeted therapies in the management of advanced gastric cancer. Overall, the report highlights the ongoing challenge of managing advanced gastric cancer and the importance of personalized treatment strategies.

## Introduction

Gastric cancer is a significant health issue worldwide, and is the fifth most common cancer and the fourth leading cause of cancer-related deaths globally [1]. It was estimated that there were approximately 1.09 million new cases of gastric cancer and over 769 thousand deaths worldwide. Despite significant advances in diagnosis and treatment, the prognosis for patients with advanced gastric cancer remains poor [2].

The standard first-line chemotherapy for metastatic gastric cancer had been consisting of fluoropyrimidines plus a platinum analog [3]. S-1 is an oral fluoropyrimidine drug including tegafur, which is a prodrug of 5-fluorouracil [4]. In our country, S-1 plus cisplatin (CDDP) had been widely used for metastatic gastric cancer patients. According to the result of the SPIRITS trial, median overall survival was significantly longer in patients assigned to the S-1 plus cisplatin arm (13.0 months) than in those assigned to the S-1 arm (13.0 vs 11.0 months, p=0.04) [5].

Salvage chemoradiation therapy has been proposed as a potential treatment option for patients with advanced gastric cancer who have failed previous treatments [6]. However, the effectiveness of salvage chemoradiation therapy in improving survival outcomes for these patients remains unclear.

Here, we present a case report of a patient with advanced gastric cancer who underwent repeated salvage chemoradiation therapy after failed initial treatments. The patient achieved long-term survival and remains disease-free several years after treatment. This case highlights the potential benefits of salvage chemoradiation therapy in selected patients with advanced gastric cancer, and the need for further studies to determine the optimal treatment approach for these patients.

## Case presentation

**Figure 1:**
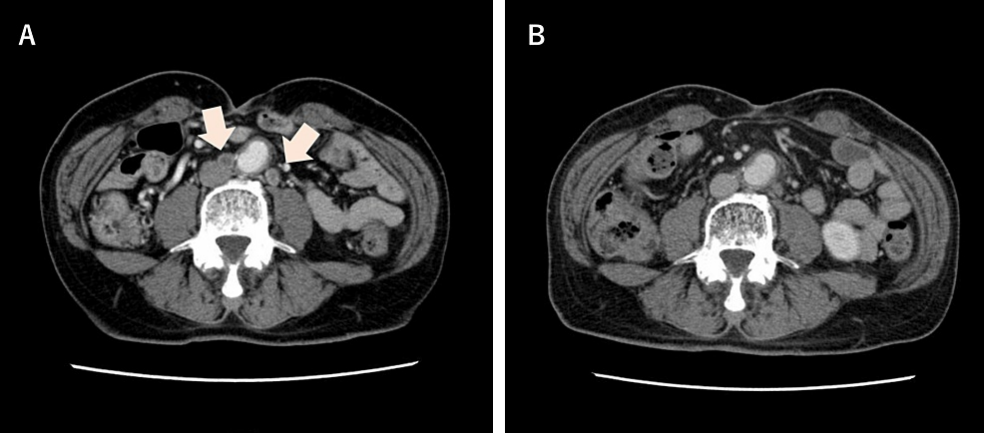
Contrast-enhanced computed tomography of the abdominal region Contrast-enhanced computed tomography of the abdominal region before 1st chemoradiotherapy (A) and after the chemoradiotherapy (B). The 0range arrow indicated swollen lymph nodes.

A 60-year-old man was referred to our hospital after adjuvant chemotherapy of oral S-1 administration for 1 year following distal gastrectomy for stomach cancer with D2 lymphadenectomy. The pathological assessment of the stomach had included non-solid type poorly differentiated adenocarcinoma with pathologically lymph node metastasis-positive (3/34). After the completion of adjuvant S-1 chemotherapy, no significant disease remained confirmed by contrast-enhanced computed tomography (CECT). However, two months later, 18F-fluorodeoxyglucose positron emission tomography-computed tomography detected para-aortic lymph node adenopathy, which was diagnosed with a recurrence of gastric cancer with a slight increase in serum carcinoembryonic antigen level of 6.5 ng/ml. He was started on chemotherapy consisting of oral S-1 (80 mg/body) administration and intravenous infusion of CDDP (88mg/body). After 5 cycles of the regimen, para-aortic lymph node adenopathy progressed, which was confirmed by CECT (Figure 1A).

**Figure 2:**
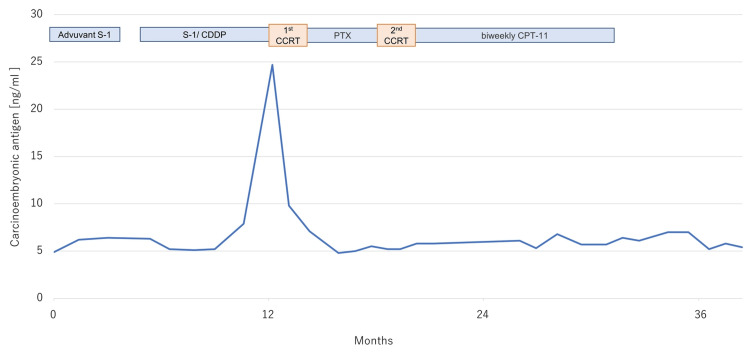
serum carcinoembryonic antigen level Time course of serum carcinoembryonic antigen level and schema of treatment modalities.

Serum carcinoembryonic antigen level rose up to 24.7 ng/ml (Figure 2).

**Figure 3:**
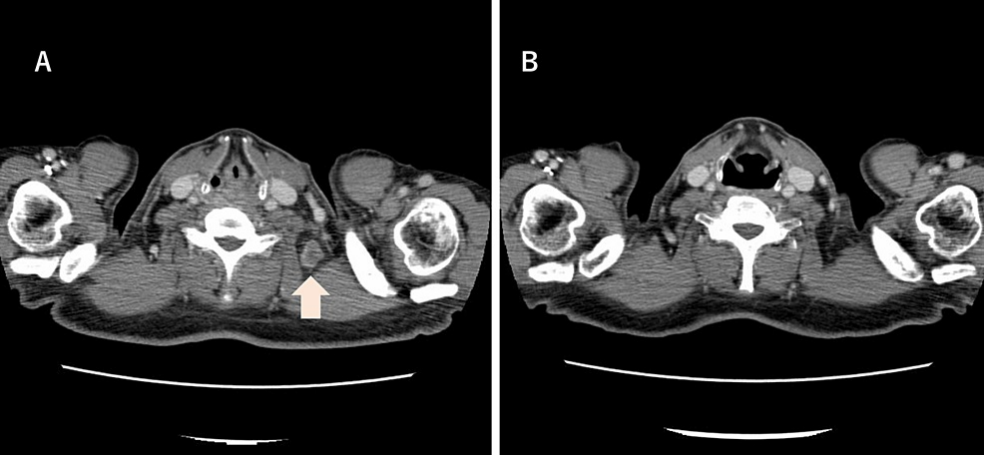
Contrast-enhanced computed tomography of the subclavicular region Contrast-enhanced computed tomography of the subclavicular region before 2nd chemoradiotherapy (A) and after the chemoradiotherapy (B). The orange arrow indicates swollen lymph nodes.

These lesions were treated with 3-dimensional conventional radiotherapy of 60 Gy in 30 fractions with concurrent chemotherapy (CCRT) of weekly paclitaxel 140 mg per body. During the CCRT, laboratory examination revealed grade 2 thrombocytopenia, grade 1 anemia, and grade 2 leukocytopenia as a treatment-related adverse event. After the chemoradiation therapy, the para-aortic metastatic lymph nodes had revealed tumor reduction confirmed by CECT (Figure 1B). However, four months later, sub-clavicular lymph node adenopathy was detected by CECT (Figure 3A).

Again, the subclavicular lymph nodes were treated with a total of 60 Gy in 30 fractions radiotherapy with concurrent chemotherapy of weekly paclitaxel 140 mg per body. Treatment-related adverse events were grade 2 dermatitis at the subclavicular area, grade 1 thrombocytopenia, grade 1 anemia, and grade 1 leukocytopenia.

After the second chemoradiation therapy, the subclavicular lymph node adenopathy regressed (Figure 3B) and the patient received biweekly irinotecan (250 mg per body) as consolidation therapy for 21 months. The patient had progression-free survival for over 5 years since the second chemoradiation therapy with no obvious adverse events.

## Discussion

In the present case, we present a patient with advanced gastric cancer who achieved long-term survival after salvage chemoradiation therapy. Despite the initial diagnosis of bulky lymph node involvement and advanced-stage disease, the patient underwent a multimodal treatment approach including surgery, adjuvant chemotherapy, and salvage chemoradiation therapy.

The optimal management of recurrent or metastatic gastric cancer remains unclear. Recently, the combination regimen with immune checkpoint inhibitors is reporting promising results. The CheckMate 649 trial demonstrated that the combination of nivolumab and chemotherapy significantly improved median overall survival and progression-free survival compared to chemotherapy alone for advanced upper gastrointestinal cancers (14.4 vs 11.1 months, p < 0.0001) [7]. The combination of nivolumab plus ipilimumab plus chemotherapy had a high response rate of 60.9%, compared to 46.9% in the chemotherapy alone group. In the ATTRACTION-4 trial, the combination regimen of nivolumab and oxaliplatin-based chemotherapy significantly improved progression-free survival (10.45 vs. 8.34 months, p=0.0007) in Asian patients with advanced or recurrent gastric or gastro-esophageal junction cancer without human epidermal growth factor receptor 2 (HER2) amplification. [8].

In addition, the identification of predictive biomarkers, such as HER2, has allowed for the development of targeted therapies in the management of advanced gastric cancer. The ToGA trial investigated the use of trastuzumab, a monoclonal antibody against HER2, in combination with chemotherapy for systemic treatment of HER2-positive advanced gastric or gastro-esophageal junction cancer [9]. This trial revealed that combination therapy significantly improved median overall survival compared with chemotherapy alone (13.8 vs 11.1 months p=0.0046). In 2020, Shitara et al. revealed that trastuzumab deruxtecan led to significant improvements in median overall survival for patients with HER2-positive advanced gastric cancer, as compared with standard therapies in DESTINY-Gastric01 trial (12.5 vs. 8.4 months, p=0.01) [10].

The management of advanced gastric cancer remains challenging, and the optimal treatment approach is still under investigation. In the present case, the patient received salvage chemoradiation therapy after the development of lymph node metastasis and subsequent metastasis to another site. The patient was initially treated with systemic chemotherapy, however, which led to a change in treatment strategy. Salvage chemoradiation therapy succeeded and achieved long-term disease control. The use of multimodal therapies might provide further benefits in the treatment of advanced disease. The present case was considered as repeat oligorecurrence, which refers to a situation where a cancer patient who has previously undergone treatment for cancer experiences a recurrence in a limited number of locations in the body [11]. Appropriate localized treatment could prolong survival in selected patients [12], while treatment consensus for oligorecurrence of gastric cancer has not been established.

## Conclusions

In conclusion, the successful management of advanced gastric cancer requires a multidisciplinary approach, surgery, chemotherapy, and radiation therapy. Localized therapy, including chemoradiation therapy for localized treatment of the secondary lesion, should also be considered in the management of the recurrent or metastatic disease.

The case presented here demonstrates the potential benefit of salvage chemoradiation therapy in achieving long-term survival in patients with advanced gastric cancer. Further studies are needed to optimize the treatment approach for these patients and to identify new therapeutic targets in the management of this challenging disease.
